# Temperature Compensation Method for Digital Cameras in 2D and 3D Measurement Applications

**DOI:** 10.3390/s18113685

**Published:** 2018-10-30

**Authors:** Marcin Adamczyk, Paweł Liberadzki, Robert Sitnik

**Affiliations:** Institute of Micromechanics and Photonics, Faculty of Mechatronics, Warsaw University of Technology, ul. Św. Andrzeja Boboli 8, 02-525 Warsaw, Poland; p.liberadzki@mchtr.pw.edu.pl (P.L.); r.sitnik@mchtr.pw.edu.pl (R.S.)

**Keywords:** camera calibration, temperature effect, 3D imaging, structured light

## Abstract

This paper presents the results of several studies concerning the effect of temperature on digital cameras. Experiments were performed using three different camera models. The presented results conclusively demonstrate that the typical camera design does not adequately take into account the effect of temperature variation on the device’s performance. In this regard, a modified camera design is proposed that exhibits a highly predictable behavior under varying ambient temperature and facilitates thermal compensation. A novel temperature compensation method is also proposed. This compensation model can be applied in almost every existing camera application, as it is compatible with every camera calibration model. A two-dimensional (2D) and three-dimensional (3D) application of the proposed compensation model is also described. The results of the application of the proposed compensation approach are presented herein.

## 1. Introduction

Digital cameras have a wide range of applications, including field detectors in three-dimensional (3D) imaging. These indispensable devices are typically characterized by high-resolution images, ease of integration and operation, high frame rate, a wide variety of available communication protocols, and a compact design. In this paper, we will focus on the application of cameras as 3D-structured light scanners [1]; but the proposed compensation method can also be easily applied to many other imaging applications.

3D-structured light scanners are routinely used in many scientific and engineering disciplines, including medicine [1,2,3] (e.g., gait analysis, skeletal spine curvature assessment, prosthetic mechanical vibrations), industry [4] (e.g., quality control, reverse engineering, precision measurements for 3D printing), or disaster management [5], in the archiving of cultural artifacts [6], and in many other domains [1]. In almost all applications, 3D scanners are expected to acquire precise measurements, with an error no greater than their declared uncertainty.

The appropriate level of precision is achieved through a careful calibration process that typically consists of at least two steps: the camera calibration process and the phase calibration process [7]. Apart from 3D measurement systems that require recalibration before each set of measurements [8], the employed measurement strategy often assumes that the camera is calibrated only once in a laboratory environment [9,10,11,12], under stabilized environmental conditions (temperature, vibration, humidity, etc.) and using specialized calibration artifacts. Once calibrated, camera-based 3D scanners are capable of stable and accurate measurements, provided that the camera and projector parameters and their relative positions remain constant. However, there is often a difference between the environmental conditions during calibration and at the location where measurements are going to be processed, e.g., measurements at archaeological excavations [13,14] or investigation scenes [4,5,9]. The implicit assumption that a scanner that is calibrated under laboratory conditions can reliably function in a completely different setting is often incorrect.

The effect of temperature on digital cameras and its adverse influence on captured images can also be observed in other applications, not only in 3D-structured light scanners. Digital cameras are often used in industrial applications to measure geometric features with high precision, up to 2 μm [15,16]. The temperature in the production hall can vary, and the temperature amplitude in a day–night cycle can also be very high. Taking into account the differences in environmental conditions, temperature is one of the most important factors that needs to be taken into account when considering the accuracy of measurements performed with any camera-based system [5,9,12,13,17,18,19,20,21]. The solution of this issue can be either software or hardware. The hardware solution is related to keeping the camera-lens system in a stable ambient temperature, by enclosing it in some kind of thermal chamber. The disadvantages of such an approach are as follows: the increased size of the camera lens system equipped with a temperature control unit and the increased power consumption. The software solution is free from these disadvantages.

Simulation of the effect of temperature on digital cameras and development of a valid temperature compensation method have been described in several works. Smith and Cope in Ref. [12] described the results of several studies on the effect of temperature on single lens reflex (SLR) camera. They focused on the temperature impact on the camera calibration parameters, especially the principal point position and the focal length. The results of their experiments show that the focal length can change by up to 0.03 mm for a temperature increase ΔT equal to 30 °C. The principal point position is also relatively strongly related to the temperature. Handel reported in Ref. [17,18,19] the outcome of studies on camera warm-up phenomena and the effect of ambient temperature changes on camera calibration. He has showed that the image drift can be up to several pixels even during the camera warm up process [17]. Similar drift can be observed as the ambient temperature changes; even small changes can cause significant changes in the captured frames [18,19]. Handel used a pinhole camera model and camera calibration proposed by Zhang in Ref. [22]. He has also proposed a mathematical model that can extend the camera calibration to include temperature compensation. His method for compensation assumes that the camera-lens system can be treated as a rigid body and that the lens is not affected by the temperature change. He also assumed that the values of the internal parameters of the camera are constant. These assumptions are very strong and our studies (see Section 3) and Refs. [20,23] show that in some cases they are not valid.

In Ref. [20], Podbreznik and Potočnik present their own method for modeling the effect of temperature on camera calibration. They also used a pin hole camera model and the calibration method proposed by Zhang [22]. They assumed that changing temperature does not affect the intrinsic camera parameters and that only translation of the camera needs to be taken into account when considering the effect of temperature. This approach is also inaccurate in some cases.

One of the most insightful studies on the effect of temperature on cameras is described in Ref. [23]. The authors presented an extensive report from their studies and proposed their own method for temperature compensation that is devoid of the previously mentioned issues. However, our studies have shown that it is not enough for only camera’s intrinsic and extrinsic parameters to be compensated. Experiments have shown that the observed pixel drift, caused by camera warm-up or changes in ambient temperature, is also dependent on the relationship between the camera-lens system and the gravitational force. The calculated compensation model for one global position of the camera becomes very inaccurate when the camera’s global position is changed; this is caused by changes in deformations resulting from the gravitational force. Another observed phenomenon is the hysteresis observed in deformations of the camera-lens system (see Section 3). Our studies, conducted at the Institute of Micromechanics and Photonics, convinced us that to obtain a valid and reliable temperature compensation model, some camera models require obligatory design modifications. Another motivation is that there is no existing method for constructing a compensation model using a camera calibration method different from that of Zhang [22] and using anything other than a pin-hole camera model.

In this paper, we present the outcome of research concerning the effect of temperature on camera calibration. We describe the results of preliminary studies that justify the need for changes in the camera design so that the camera behavior becomes more predictable and can be described using a mathematical model. We demonstrate such a modified camera design. We also propose a software temperature compensation method for digital cameras. These studies are the continuation of research introduced in Ref. [9], where we described the effect of temperature on the calibration accuracy of a 3D-structured light scanner and proposed a method for its compensation.

## 2. The Test Stand

We conducted the previous research (described in Ref. [9]) with access to a professional thermal chamber. The chamber had a working volume of 4×3×3 m and supported control of temperature within a range of −40 °C to 70 °C ± 0.5 °C with a forced air flow within a range of 0.5 m/s to 4 m/s ± 0.01 m/s and a relative humidity of 10% to 90% ± 2%. Access to this chamber was limited; the operating cost is very high and we do not require such a large operating volume. Hence, we decided to build our own thermal chamber. For this purpose, we used a freezer, in which we have installed a heater unit that can be controlled by a PID regulator with two degrees of freedom (one for heating and one for cooling so that we can control the inner temperature over a wide range). As a heater, we have used an electric air heater (power: 2000 W) that is controlled by a pulse-width modulation (PWM) controller.

**Figure 1:**
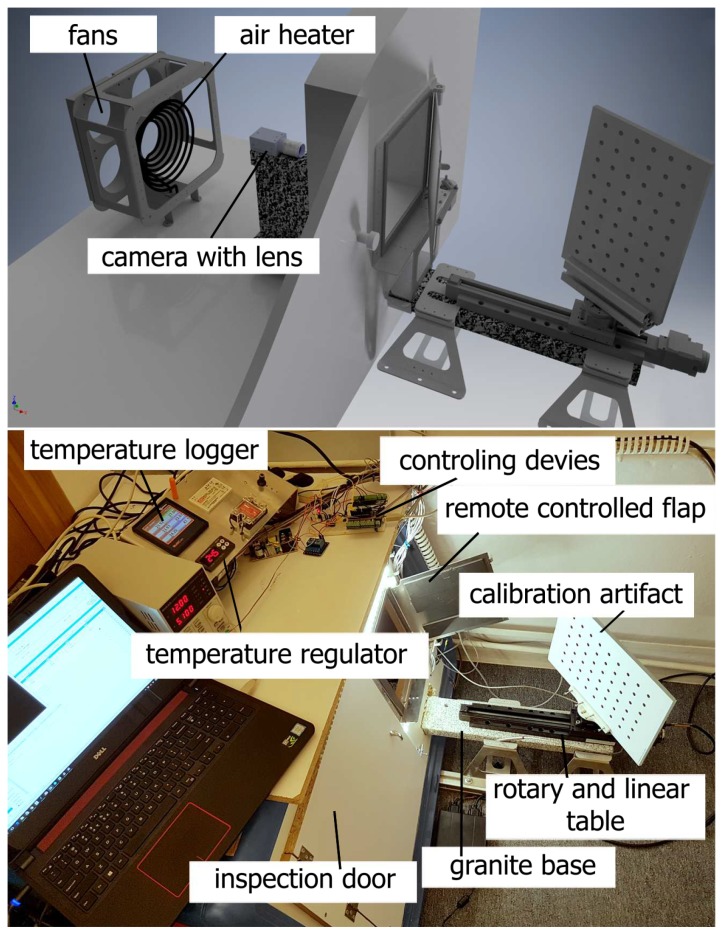
The designed thermal chamber and the test stand, demonstrating the experimental setup.

The designed thermal chamber has inner dimensions of 1200×90×80 mm and can regulate the temperature over a range of −10 °C to + 70 °C (Figure 1). It is equipped with an electronically driven inspection flap that is normally closed, but can be opened remotely. This allows us to conduct measurements with the camera calibration artifact placed outside the thermal chamber—the artifact is not exposed to varying ambient temperature. The chamber is also equipped with a passive air dehumidifier to prevent the condensation of water vapor.

The test stand is designed so that the tested camera and the calibration artifact are mounted on one mono-block of granite (with a very low temperature expansion coefficient α= ~$5.0\times 10^{-6}$ [$K^{-1}$]). One side of the granite slab is located inside the thermal chamber, while the other side is outside the chamber. Using Edlén’s formula [24] and Snell’s law for the designed geometry of the test stand, the maximal value of error caused by the changes in air refraction index (the refraction of light at the interface between two media of different refractive index: air with different temperature) is calculated to be no greater than 4 μm for a temperature difference of $\Delta T=30{\;}^{{}^{\circ}}$C. The value of this error is negligible compared with errors observed during experiments (the distance between two neighboring pixels on the image plane is equal to 114 μm on the calibration artifact plane, and the calculated error is the maximum near the borders of each captured frame).

This construction allows us to treat the camera support (also made from granite) and the calibration artifact as a rigid body, where the effect of deflections caused by changes in temperature is negligible. There are five temperature sensors that are used in the test stand: one is attached to the inner part of the granite base, another to the outer part, one to the calibration artifact, one to measure the air temperature near the camera, and one to measure the temperature inside the laboratory. We decided not to mount any temperature sensor on the camera because it could disturb the camera deflection process caused by thermal expansion/shrinking. To detect the camera temperature, we used the internal temperature camera sensor (if available) and thermal imaging camera FLIR E40 [25].

**Figure 2:**
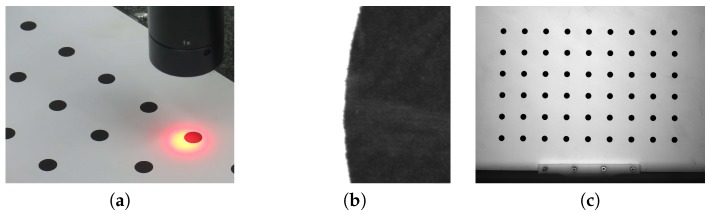
The calibration artifact. (**a**) The validation process of the calibration artifact. (**b**) The RAW image from the Carl Zeiss Viscan Optical Header representing the magnitude of one of the markers, where the size of the measurement field is 4.8×6 mm and resolution is 6 μm. (**c**) The calibration image captured by the tested camera, 6×9 circle markers with a diameter of 8 mm and nominal distances along the *X* and *Y* axes of 30 mm.

To detect the image drift, we used the a calibration artifact: a 9×6 matrix of black circle markers (the first marker is located on the bottom left) printed on a matte white foil stuck to a glass plate. This artifact was previously validated on a coordinate measurement machine (CMM) equipped with a Carl Zeiss Viscan Optical Header (Figure 2) so that the precise coordinates of the center of every marker were determined.

The calibration artifact was attached to a rotary-linear table (linear table Standa 8MT160-300, with a 300-mm travel range and resolution up to 0.31 μm [26], rotary table Standa 8MR151 with resolution up to 0.6 arcmin [27]). This setup allows for conducting automatic camera calibration and subsequent 3D validation of our compensation model. The camera was controlled by a PC computer. If the camera supported an internal temperature sensor, each captured frame also had the temperature information (as a separate file saved with the frame). To obtain the coordinates of the center of every marker, we implemented a sub-pixel algorithm in MATLAB, that is resistant to thermal noise and can provide valid results for a wide variety of ambient lighting and marker sizes [4,7,28].

## 3. Preliminary Studies

### 3.1. Warm-Up Effect

**Figure 3:**
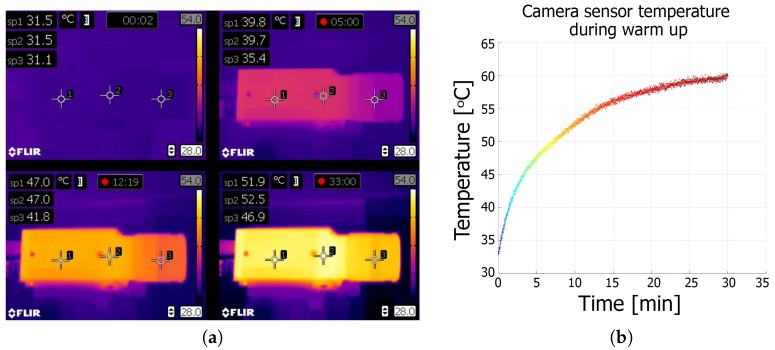
The warm-up effect of the tested camera. (**a**) The distribution of temperature on the surface of the tested camera (PointGrey Grasshopper2) and lens (Fujinon HF25SA1B) observed with a thermal imaging camera. (**b**) The temperature measured by the camera sensor as a function of time.

**Figure 4:**
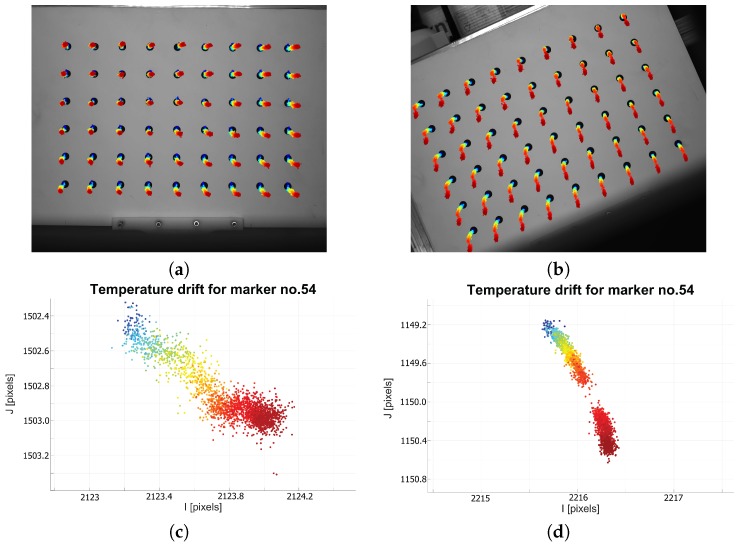
The observed image drift for normal (**a**) and rotated (**b**) camera (Pointgrey Grasshopper 2) positions. The background image is the first captured frame. Colored dots (blue for reference temperature—the coolest) represent the next location of calculated marker centers. Each subsequent coordinate was multiplied by a factor of ×100 for better visualization. Graphs (**c**,**d**) represent the temperature drift, in normal (**c**) and rotated (**d**) camera positions, for marker no. 54.

**Figure 5:**
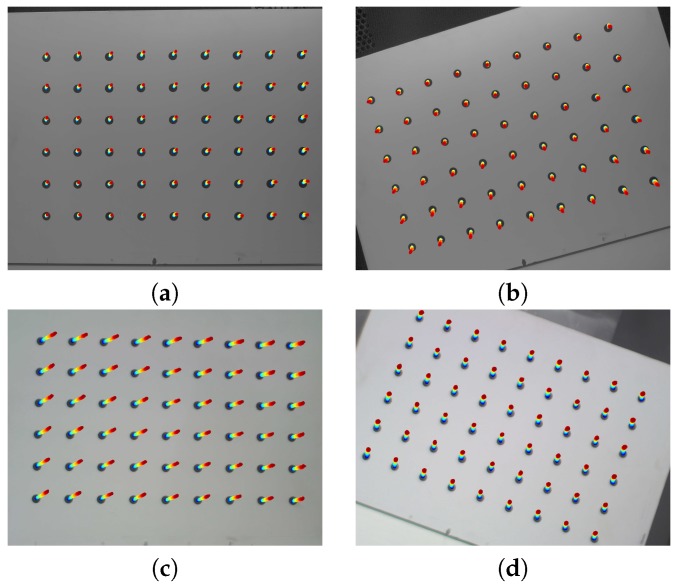
The image drift observed for normal (**a**,**c**) and rotated (**b**,**d**) camera positions. The background image is the first captured frame. Colored dots (blue for reference temperature—the coolest) represent the next locations of calculated marker centers. Each next coordinate was multiplied ×100 times for better visualization. Figures (**a**,**b**) present image drift for the IDS camera; (**c**,**d**) are the image drift for the Basler camera.

The first experiment performed involved an observation of the camera warm-up process. For this experiment, we used three different camera models, which included a CCD Pointgrey Grasshopper2 camera with a 5.0 MP sensor size (GS2-GE-50S5M-C) [29] with a small lens from Fujinon HF25SA-1B [30]; a CMOS Basler asA2500 [31] with a C125-1218-5M lens [31]; and a CCD IDS UI-6280SE-M-GL Rev 3 IDSCamera with a high-quality Tamron M23FM12 lens [32]. We fixed each camera and lens assembly to a granite support and initiated frame capture immediately after the camera was switched on. The internal temperature of the thermal chamber was the same as the ambient room temperature ($24{\;}^{{}^{\circ}}$C), which remained constant throughout the experiment. The flap was left open to allow for continuous frame capture, and the time interval between frames was set to 500 ms, with the shutter set to 100 ms. We captured ~3600 frames and logged the sensor temperature for each frame (only for the PtGrey camera; the Basler and IDS camera models do not support an internal temperature sensor). The coordinates of each dot marker center were then extracted from each frame, and this information was used to monitor the image drift. Simultaneously, the surface temperature of the test camera was measured using the thermal imaging camera (we defined three measuring points on the lens, the camera housing near the sensor, and the back of the camera). The experiment was repeated with the camera rotated along its lens axis by $20^{{}^{\circ}}$ (after the camera cooled down). The results of this experiment are presented in Figure 3, Figure 4 and Figure 5.

Based on the preliminary experimental results, we can conclude that the camera and lens cannot be treat as a rigid body. The only source of heat during the experiment was the camera. The temperature of the camera body increased by ~$20{\;}^{{}^{\circ}}$C, while the temperature of lens increased by ~$16{\;}^{{}^{\circ}}$C (Figure 3) A mean drift of 0.3 pixels for the horizontal and 1.1 pixels for the vertical image coordinates was observed. This drift is caused by the warm-up effect and is also dependent on the camera’s spatial positioning. When the camera is oriented in space (a real-world equivalent scenario, is the tilting of a 3D SL scanner to acquire images of an inaccessible feature), the trajectories of the observed drift are affected. This phenomenon was observed in every tested camera model (see Figure 4 and Figure 5). We therefore conclude that the orientation of the camera is an important factor when considering the effect of drift, in addition to and separate from the warm-up effect.

### 3.2. Tests in Thermal Chamber

**Figure 6:**
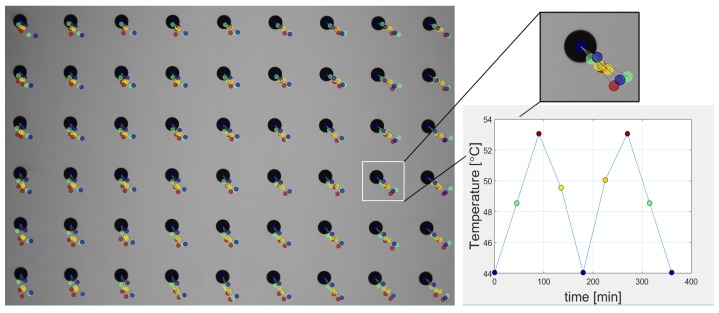
The image drift (multiplied ×100) observed during changes in the ambient temperature and the temperature measured by the internal camera sensor.

Additional tests were conducted that focused on describing the behavior of the camera-lens system under changing ambient temperature. The setup was the same as in the previous experiment, except that the ambient temperature was systematically changed. The camera (Pointgrey Grasshopper 2 [29]) was turned on for ca. 45 min, and the experiment was initiated once thermal equilibrium was achieved. One frame was captured at a time for each preset temperature inside the thermal chamber. The time interval between each measurement was approximately 45 min. This ensured that the camera was allowed to reach thermal equilibrium after each new temperature setting. The flap was closed during the experiment, except during frame capture. The starting ambient temperature ($24{\;}^{{}^{\circ}}$C) was used as the reference temperature. Two measurement cycles were performed, as the ambient temperature was increased and subsequently decreased. The calculated image drift is presented in Figure 6.

The results indicate that the image drift caused by the camera’s (Pointgrey Grasshopper 2.0 [29]) deflections, during the increase and decrease in the ambient temperature, is heterogeneous. The observed marker coordinates did not reach their final values relative to the reference temperature; hence, hysteresis was observed. We repeated the experiment several times to attempt to characterize the hysteresis, but the behavior was unpredictable. This response is likely due to relaxation of thermal tensions that accumulated in various regions of the camera and lens. A similar lack of repeatability was observed for the other camera models. As in the first experiment, the observed image drift was unpredictable, making it difficult to fit a valid compensation model to this data.

## 4. Camera Modifications

Our preliminary studies indicate that the behavior of a camera that is exposed to varying temperature is quite complex. In addition to changes to the internal camera parameters, deflections of the camera body and lens are also observed. This situation is further complicated by the fact that the system’s response is irregular and the observed hysteresis is unpredictable (Figure 6). To effectively address these problems, the original camera was reassembled and modified. The modifications took into account our conclusion that the crucial sources of concern were the lens C-mount and the support of the sensor that was directly connected to the camera housing.

**Figure 7:**
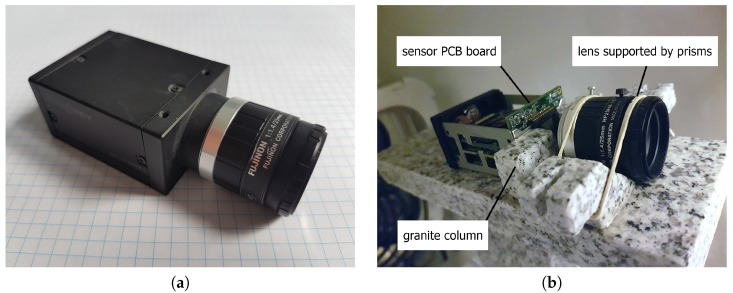
The original and modified camera unit. (**a**) The original camera unit. (**b**) A photo of the modified camera. The lens is positioned on two granite prisms while the sensor is supported between two granite columns and can only translate along the vertical sensor axis and rotate around the horizontal sensor axis.

We therefore removed the entire camera housing, fixed the camera sensor housing between two small granite columns, and detached the lens from the camera body. The lens was instead positioned on two granite prisms and supported using an elastic mounting kit. This ensured that the camera sensor had only two degrees of freedom-translation along the vertical sensor axis, and rotation around the horizontal sensor axis. There were no direct mechanical connections between the camera and the lens. The camera modifications are illustrated in Figure 7.

We decided to modify only the Pointgrey Grasshopper 2.0 [29] camera model, as it was the only one equipped with an internal temperature sensor.

## 5. Experiment with Modified Camera Unit

**Figure 8:**
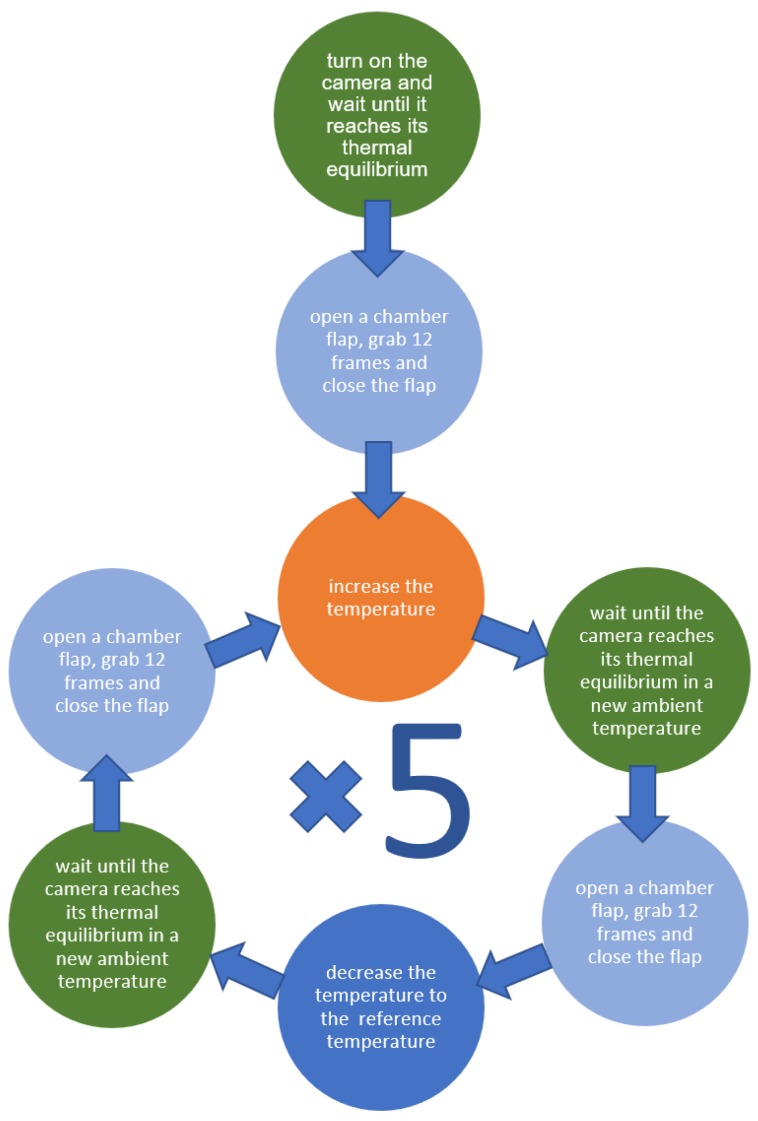
Workflow for the experiment with the modified camera unit. The temperature was increasing gradually, starting from the reference temperature. The aim was to observe the presence of any hysteresis associated with the camera’s thermal drift.

The behavior of the modified camera unit was tested in a thermal chamber. After positioning the unit in the chamber, the ambient temperature was slowly increased. The experiment was organized as shown in Figure 8.

**Figure 9:**
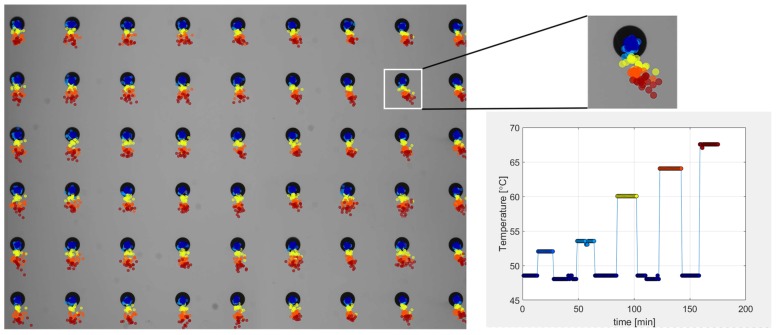
Observed image drift (multiplied ×100) during changes in the ambient temperature; and the measured temperature of the internal camera sensor of the modified camera unit. The hysteresis in the image drift is no longer observed.

The results of these experiments are presented in Figure 9. We observed more predictable behavior of the camera during cooling and warming, and the hysteresis effect was not evident. Other tests (warming up in normal and rotated camera positions) were performed, and the results indicated that the orientation of the camera did not affect the observed image drift.

The final experiment involved grabbing frames in the extended range of the ambient temperature (from $10{\;}^{{}^{\circ}}$C to $52{\;}^{{}^{\circ}}$C) to validate the compensation model.

## 6. Compensation

### 6.1. The Compensation Model

**Figure 10:**
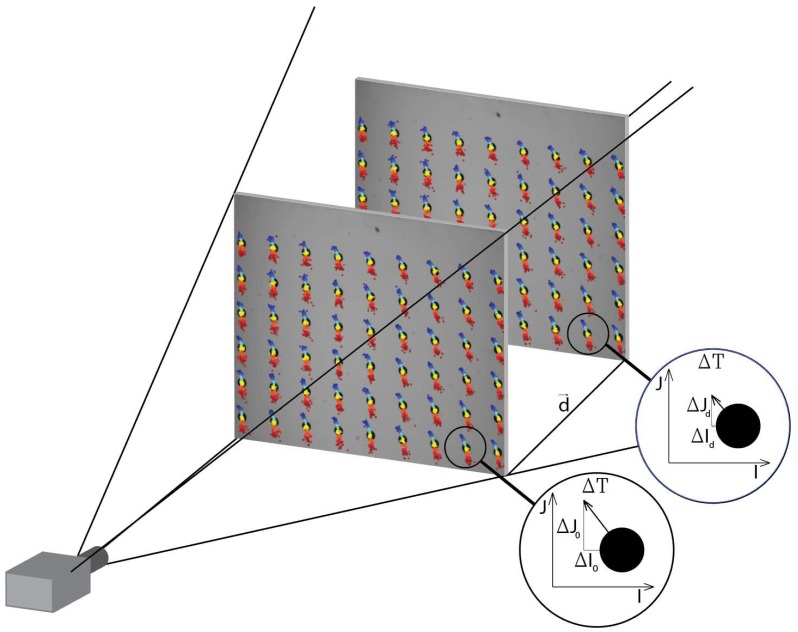
The deviations $\Delta I_{0}$ and $\Delta J_{0}$ caused by temperature drift ΔT will cause a change in $\Delta I_{d}$, $\Delta J_{d}$ for every position *d* of the calibration artifact. The compensation proposed for 2D images is valid for the entire field of view of the camera.

In most 3D scanning applications that require camera calibration, a pinhole camera model and Zhang’s method for determining intrinsic and extrinsic values [22] or a real camera model [7] is used. The use of a pinhole camera model is justified because of its wide implementation (e.g., OpenCV camera calibration algorithm [33], MATLAB Camera Calibration [34]) and reliable results [35]. For more precise applications, a real camera model [7] may be implemented, as this facilitates lower measurement errors (e.g., a low value of the maximal permissible error $E_{MPE}$) [36]) for a 3D-structured light scanner [4,5]. In addressing the thermal issues associated with the camera, we attempted to develop a compensation model that could be applied to a wide range of camera models and calibration approaches. As such, a new approach was proposed that utilizes 2D images. Such a model is valid not only for the plane with the calibration artifact, but also for the entire field of view of the camera (see Figure 10).

The independent variables utilized in the compensation model include the set of calculated marker center coordinates $I_{unc}$ and $J_{unc}$ for various ambient temperatures of the camera coordinate system. The dependent variables are the calculated centers of the dot markers $I_{ref}$ and $J_{ref}$ for the reference temperature. The output functions are an *n*-th degree polynomial $P^{n}$, which describes the image drift caused by the varying ambient temperature. We used a polynomial fit to determine the coefficients associated with the model [37].

$$I_{compensated}=P^{n}\left(I_{uncompensated},J_{uncompensated},T\right)$$

$$J_{compensated}=P^{n}\left(I_{uncompensated},J_{uncompensated},T\right)$$

### 6.2. Thermal Compensation

**Figure 11:**
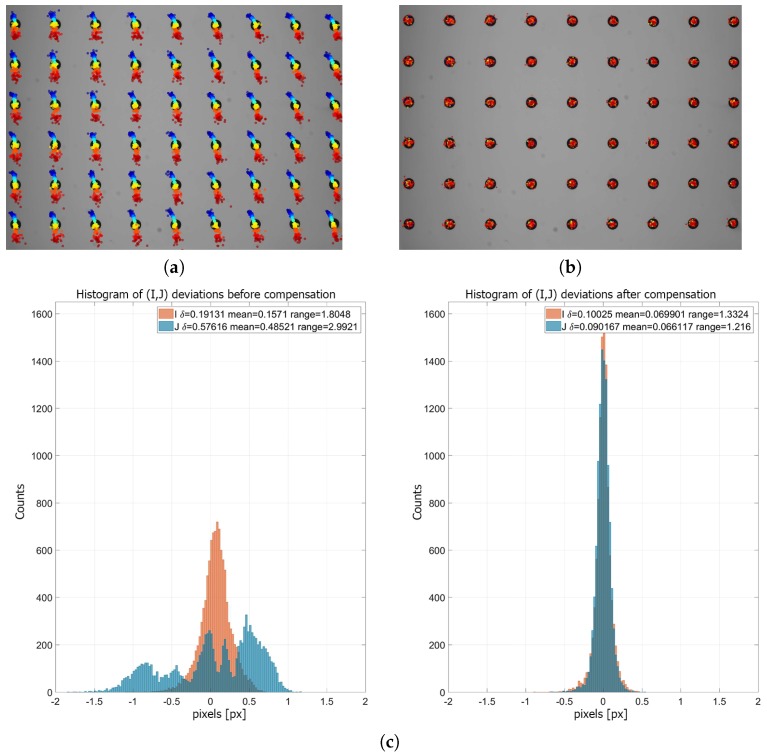
Compensation results. (**a**) Calculated thermal drift (multiplied ×100) for the marker centers used to prepare the compensation model. (**b**) Result of compensation and (**c**) histogram of deviation of coordinates, before and after compensation (fourth-degree polynomial).

The best fitting results were observed using a fourth-degree polynomial. Figure 11b shows the results when applying compensation to a series of frames that were grabbed over a range of temperature values (not the same temperatures used for building the model). The histogram of the deviation result is presented in Figure 11c. We determined that the values for the RMS error for the *I* and *J* coordinates, before and after compensation, were $\delta I_{unc}=0.191$ px, $\delta J_{unc}=0.576$ px and $\delta I_{com}=0.100$ px, $\delta J_{com}=0.090$ px, respectively, as indicated in Figure 11c.

**Figure 12:**
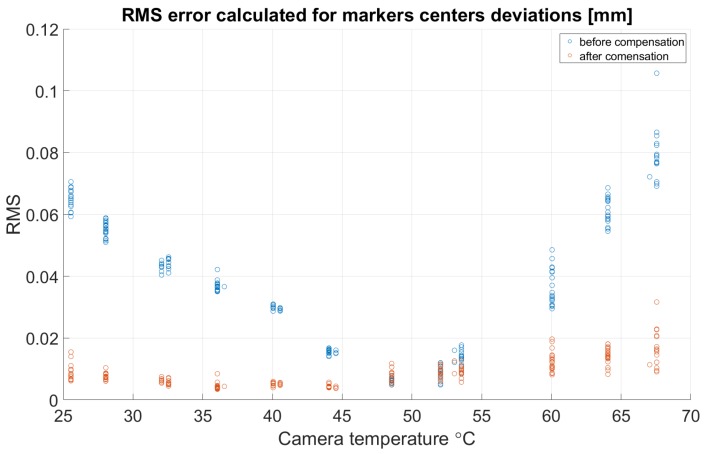
The RMS error calculated for marker center deviations before and after compensation.

As long as the proposed compensation method is prepared on 2D images and the compensation model is based on only one calibration artifact position, we do not need to calculate camera calibration error (e.g., reprojection error). To represent the compensation results in metric units, we recalculated each dot marker center coordinate from pixels to millimeters (this can be performed because the real dimensions of the calibration artifact are known; see Section 2 and Figure 2). For each grabbed frame at each temperature, the deviations between each marker coordinates and the same marker coordinates at the reference temperature were calculated. Then, for each grabbed frame, the RMS error was estimated. In Figure 12, the RMS error as a function of temperature is presented.

**Figure 13:**
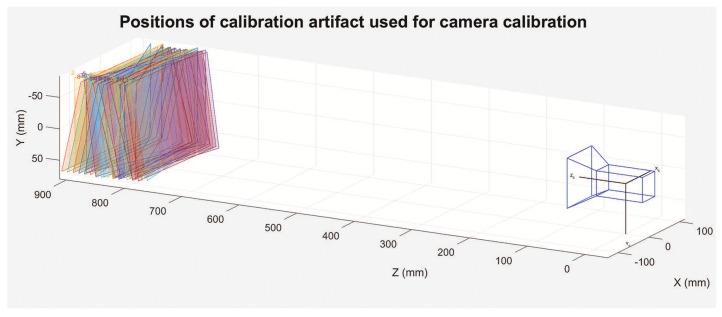
Positions of the calibration artifact used for camera calibration. A real camera model was used, and the calibration method is described in Ref. [7].

**Figure 14:**
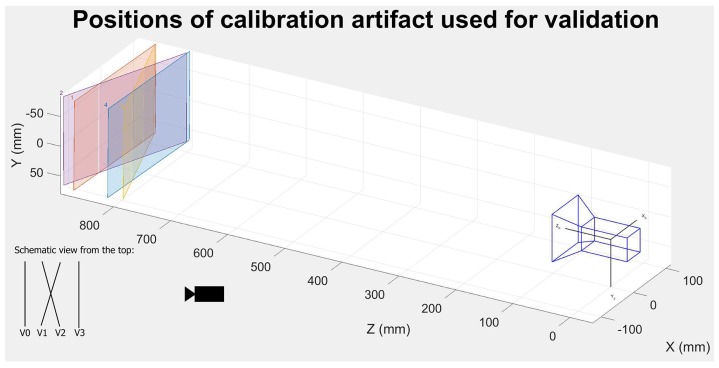
Positions of calibration artifact V0–V3 used for validation of the proposed thermal compensation model.

The final test involved performing a camera calibration at a reference temperature and determining whether or not the proposed mathematical model could effectively compensate for the effect of temperature. Using precise linear and rotary tables [26,27], we positioned a calibration artifact in 34 different positions and collected data for calibration as shown in Figure 13. A real camera model and the camera calibration method described in Ref. [7] were utilized. To evaluate the quality of the compensation model, an additional four frames were grabbed at 12 different temperatures. For each temperature, we positioned the calibration artifact at four different validation positions V0 to V3 by shifting and rotating the artifact, as shown in Figure 14, using a previously documented linear and rotary table [26,27]. We were careful to verify the repeatability of the artifact’s position for each temperature. The camera calibration performed at the reference temperature was used to calculate the exact transformation between each artifact positions V0 to V3, for each temperature. A comparison of the transformations obtained before and after compensation facilitated an evaluation of the efficacy of the proposed compensation model.

## 7. Results

**Figure 15:**
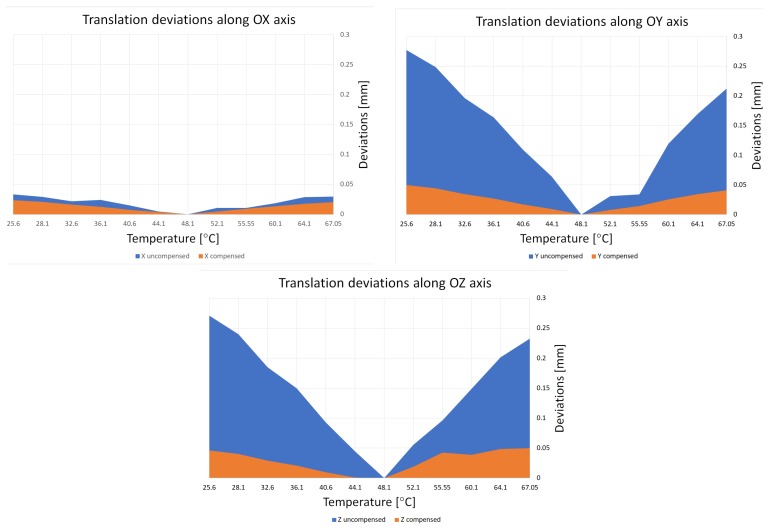
Average deviation of translations of artifact position, calculated for 11 different temperature values, relative to the artifact position calculated at the reference temperature along the *X*, *Y*, and *Z* axes, before (blue color) and after (orange color) compensation.

The results obtained using the compensation model are presented in Figure 15 and Table 1. The presented graphs illustrate the average deviation (absolute values) of translation along the *X*, *Y*, and *Z* axes, for artifact positions from V0 to V3 (we present only the translation because the effect of temperature on rotation was negligible). These values were calculated using the actual temperature, relative to the calculated position for the reference temperature, both before and after compensation. In Table 1, the calculated and averaged values of this deviation are presented. Results are presented for only two different temperatures, as additional data would not present any new information.

The average translation deviation during the investigated temperature range, before and after compensation, was calculated as follows:

- along the *X* axis: before compensation 0.0177 mm, after compensation 0.0124 mm,
- along the *Y* axis: before compensation 0.1343 mm, after compensation 0.0254 mm,
- along the *Z* axis: before compensation 0.1420 mm, after compensation 0.0287 mm.

## 8. Discussion and Conclusions

The results presented in Figure 15 and Table 1 demonstrate that the temperature drifts along the *Y* and *Z* axes are dominant. This phenomenon is related to the manner in which the camera modifications were performed, given that the camera sensor was fixed between two granite columns. This significantly limited the range of movement of the camera sensor resulting from variations in the ambient temperature. The most likely directions of movement were translation along the sensor’s vertical axis and rotation along its horizontal axis. We believe that this is the reason why the drift along the *Y* axis is significantly greater than that along the *X* axis. The calculated artifact deviation of position for the *Z* axis is most likely caused by a combination of effects, including the translation of the sensor along its vertical axis, rotation along its horizontal axis, and stretching/shrinking along the sensor’s horizontal axis. The stretching and shrinking was the result of geometrical imperfections of the granite columns.

It is important to note that the proposed compensation model significantly reduced the calculated artifact transformation deviation. The 2D compensation results shown in Figure 15 and Table 1 indicate that the proposed method can improve camera calibration and can also be used for 3D measurements. Improvements of 29%, 81% and 80% were observed along the *X*, *Y*, and *Z* directions, respectively. To better understand the compensation result, we can consider an object placed in front of a camera, at a distance of 1 m. The camera-lens system is selected such that 1 pixel is equal to 0.1 mm. When the temperature increases by $+5{\;}^{{}^{\circ}}$C, the calculated object positions will be affected by thermal error with a value of approximately 0.09 mm. However, when the ambient temperature is increased by $+20{\;}^{{}^{\circ}}$C, the induced thermal error can be as high as 0.34 mm. When the compensation model was applied, the calculated errors were no greater than 0.05 mm in both cases.

It is clear from previously published results that the impact of temperature on camera calibration is a very complex issue. In particular, the response of a standard camera unit that is exposed to varying ambient temperature is unpredictable. There is a need for modification of the camera unit design to address this problem. A new design should improve the device’s stability and repeatability and significantly minimize temperature-induced drift in acquired images. We have proposed a method for performing such modifications. Our results indicate that the images acquired from the modified camera are much more repeatable when the camera is exposed to varying ambient temperatures, and in general, it is possible to perform thermal compensation. Our camera design is only a prototype, built for laboratory experiments. It is difficult to imagine the use of such a camera unit in a real measurement device. In a real application, more appropriate materials like invar [38,39], Cer-Vit [40,41], or zerodur [41] should be considered.

Our approach to thermal compensation is based on the acquisition of 2D images; however, we have shown that the method is valid for 3D space. Previous compensation methods [19,20,23] are position dependent and are therefore ineffective if the camera’s position changes during or after the calibration process. In our approach, the final result is valid for every camera orientation. Thus, the presented compensation method is a significant improvement over many of the contemporary camera calibration techniques.

## Figures and Tables

**Table 1:** Translation deviation for calibration artifact positions V0–V3 before and after compensation.

Translation Deviation [mm]							
Temp.	Pos.	Before Compensation			After Compensation		
		X	Y	Z	X	Y	Z
55.55	V0	0.0171	0.0289	0.1174	0.0093	−0.0034	0.0354
	V1	−0.0093	0.0193	0.2016	−0.0139	−0.0081	0.0341
	V2	0.0000	0.0537	0.0174	−0.0041	0.0312	−0.0761
	V3	0.0157	0.0341	0.0481	0.0092	0.0150	−0.0244
	aver (abs)	0.0105	0.0340	0.0961	0.0091	0.0144	0.0425
67.05	V0	−0.0366	−0.1943	0.1911	0.0026	−0.0058	−0.0156
	V1	−0.0129	−0.2312	0.3267	0.0327	−0.0121	0.1198
	V2	−0.0352	−0.2235	0.2352	0.0228	0.0592	0.0187
	V3	−0.0344	−0.2015	0.1775	0.0234	0.0873	−0.0455
	aver (abs)	0.0298	0.2126	0.2326	0.0204	0.0411	0.0499
